# ERAS thyroidectomy within the Italian DRG-based public health system: surgical efficiency is penalized

**DOI:** 10.1007/s13304-026-02698-4

**Published:** 2026-06-03

**Authors:** Francesco Brucchi, Carla Colombo, Pietro Paolo Bianchi, Giuseppe Mercante, Diego Barbieri, Davide Lombardi, Giuseppe Spriano, Mario Bussi, Leone Giordano, Luigi Boni, Piergiorgio Danelli, Luca Persani, Gabriele Materazzi, Paolo Miccoli, Renzo Dionigi, Gianlorenzo Dionigi

**Affiliations:** 1 Department of Pathophysiology and Transplantation, University of Milan, Milan, Italy; 2 Division of General Surgery, Istituto di Ricovero e Cura a Carattere Scientifico (IRCCS) Istituto Auxologico Italiano, Milan, Italy; 3 Division of Endocrinology, Istituto di Ricovero e Cura a Carattere Scientifico (IRCCS) Istituto Auxologico Italiano, Milan, Italy; 4 Department of Health Sciences, San Paolo Hospital, University of Milan, 20142 Milan, Lombardy Italy; 5 Department of Otolaryngology-Head and Neck Surgery, IRCCS Azienda Ospedaliero-Universitaria Di Bologna, Bologna, Italy; 6 Alma Mater Studiorum-Università di Bologna, Bologna, Italy; 7 Division of Head and Neck, Otorhinolaryngology Unit, San Raffaele Scientific Institute IRCCS (Istituto Di Ricovero E Cura a Carattere Scientifico), Milan, Italy; 8 School of Medicine, Vita-Salute San Raffaele University, Milan, Italy; 9 Unit of Otorhinolaryngology-Head and Neck Surgery, Department of Medical and Surgical Specialties, Radiologic Sciences and Public Health, ASST Spedali Civili di Brescia, University of Brescia, Piazzale Spedali Civili 1, 25123 Brescia, Italy; 10 Otorhinolaryngology - Head & Neck Surgery Unit, IRCCS Humanitas Research Hospital, Milan, Italy; 11 Department of Biomedical Sciences, Humanitas University, Milan, Italy; 12 Department of General and Minimally Invasive Surgery, Fondazione IRCCS - Ca’ Granda - Ospedale Maggiore Policlinico di Milano, University of Milan, Milan, Italy; 13 General Surgery Department, Luigi Sacco University Hospital, Via G. B Grassi 74, 20157 Milan, Italy; 14 Endocrine Surgery Unit, Department of Surgical, Medical, Molecular Pathology and Critical Area, University Hospital of Pisa, 56126 Pisa, Italy; 15 University of Pisa, Pisa, Italy; 16 University of Insubria, Varese, Italy

**Keywords:** Thyroidectomy, Fast-track surgery, Day surgery, Enhanced recovery after surgery, ERAS, Hypocalcemia, Hospital readmission, Cost-effectiveness, Health economics, DRG reimbursement, Italian national health service, Endocrine surgery

## Abstract

Enhanced Recovery After Surgery (ERAS) pathways safely reduce length of stay (LOS) and resource use in many surgical fields, but their economic impact in thyroidectomy within the Italian National Health Service (SSN) is unclear, particularly under Diagnosis-Related Group (DRG) rules that penalize early discharge. A decision-analytic model compared an ERAS-inspired thyroidectomy pathway—same-day discharge after hemithyroidectomy and 24-hour discharge after total thyroidectomy—with a conventional DRG-driven pathway based on ≥2 postoperative inpatient days. The analysis adopted a hospital/provider perspective over 30 days, using data from a high-volume endocrine surgery unit and contemporary ERAS literature. Outcomes included LOS, postoperative complications, 30-day readmissions, direct hospital costs, and contribution margin under current SSN tariffs (including early-discharge penalties) and under a neutral reimbursement scenario. In hemithyroidectomy (400 cases/year), ERAS reduced mean LOS from 2.6 to 0.33 days (–2.27 days; *p*<0.001), freeing 908 bed-days annually and lowering variable costs by €1,332 per patient, without increasing complications or readmissions. Despite a 30% DRG penalty for LOS <2 days, the contribution margin increased by €147 per case. In total thyroidectomy (600 cases/year), ERAS reduced LOS from 3.1 to 1.16 days (–1.94 days; p<0.001) and variable costs by €1,079 per patient, again without compromising safety. However, DRG penalties reduced the margin by €481 per case, resulting in an annual loss of approximately €289,000 despite substantial real-resource savings. Overall, ERAS reduced mean LOS by 2.1 days and variable costs by €1,190 per patient across 1,000 procedures. ERAS thyroidectomy is clinically safe and markedly reduces LOS and hospital costs, but current SSN DRG rules penalizing early discharge blunt or reverse its financial benefits for public hospitals, particularly for total thyroidectomy. These findings highlight a structural mismatch between surgical efficiency and reimbursement incentives and support revising thyroidectomy tariffs to reward evidence-based early discharge. Because this is a model-based economic evaluation, these results should be interpreted as scenario-based evidence rather than as definitive real-world cost-accounting estimates.

**Supplementary Information:**

The online version contains supplementary material available at https://doi.org/10.1007/s13304-026-02698-4.

## Introduction

Enhanced Recovery After Surgery (ERAS) programs integrate evidence-based, multimodal strategies to shorten recovery, reduce complications, and lower costs without compromising safety [1, 2]. Their adoption has generated substantial economic benefits in general surgery, where reduced length of stay (LOS) directly translates into lower hospital costs and improved resource utilization [3]. In thyroid and parathyroid surgery, however, the implementation of ERAS principles remains inconsistent, and their economic implications have not been systematically evaluated [4–13].

Thyroidectomy is particularly well suited to ERAS pathways, given its relatively short operative time, low postoperative pain, and limited risk of severe complications in appropriately selected patients [4]. Nevertheless, traditional two- to three-day hospitalizations are still common, largely driven by concerns regarding postoperative hemorrhage and hypocalcemia rather than robust cost–benefit evidence [14]. As a result, potentially avoidable inpatient days persist despite growing evidence supporting the safety of early discharge protocols [4].

In Italy, this gap between evidence and practice is amplified by the reimbursement structure of the Servizio Sanitario Nazionale (SSN) [15–17]. Under the current Diagnosis-Related Group (DRG) system, short hospital stays may trigger reduced reimbursement when LOS falls below regulatory thresholds, even in uncomplicated cases (Supplementary Table 1) [17]. This creates a structural paradox in which ERAS pathways that reduce real resource consumption and optimize bed utilization may paradoxically result in financial disadvantages for public hospitals. Conversely, private and insurance-based providers, operating under less restrictive reimbursement schemes, have more readily adopted early discharge models, demonstrating both clinical feasibility and economic sustainability.

This misalignment between reimbursement incentives and ERAS-driven efficiency has relevant implications for equity, resource allocation, and the long-term sustainability of public surgical services. However, few studies have explicitly quantified the economic consequences of ERAS thyroidectomy under different funding frameworks or explored how tariff structures may influence the adoption of evidence-based organizational innovations [4–13]. Importantly, ERAS should not be equated with “short-stay” surgery: whereas a short stay can be achieved within a conventional care model simply by discharging patients earlier, ERAS refers to a structured, standardized pathway that integrates preoperative, intraoperative, postoperative, and discharge/follow-up components, of which early discharge is a downstream consequence rather than the defining feature.

To address this gap, we developed a decision-analytic model to evaluate the clinical and economic impact of an ERAS-based thyroidectomy pathway, combining same-day discharge after hemithyroidectomy and 24-hour discharge after total thyroidectomy, compared with conventional multi-day hospitalization. By integrating clinical outcomes with detailed cost modeling under both SSN and neutral reimbursement scenarios, this study aims to clarify whether ERAS-inspired thyroid surgery can deliver higher value through reduced resource use and improved organizational efficiency.

## Methods

### Study design and perspective

This was a model-based economic evaluation comparing an ERAS-inspired thyroidectomy pathway with a conventional Diagnosis-Related Group (DRG)–driven hospitalization pathway within the Italian National Health Service (Servizio Sanitario Nazionale, SSN) [15–17]. The analysis adopted a hospital/provider perspective, capturing direct medical costs borne by the treating institution under current DRG reimbursement rules, and was conducted in accordance with CHEERS 2022 recommendations [15, 16, 18].

### Clinical pathways and population

The ERAS pathway reflected a standardized protocol implemented in a high-volume endocrine surgery unit, with same-day discharge after hemithyroidectomy (approximately 6 h of observation) and routine 24-hour discharge after total thyroidectomy in eligible patients [4–13] (Supplementary Table 2). The comparator represented usual care in Italian public hospitals, characterized by conventional multi-day hospitalization (≥ 2 postoperative days) aligned with DRG requirements for full reimbursement [15–17]. Central or lateral neck dissection, redo surgery, minimally invasive approaches, and pediatric cases were excluded to avoid confounding by case complexity.

In our institution, the ERAS thyroidectomy pathway is a structured, multimodal protocol rather than a simple short-stay or fast-track model. It combines standardized preoperative patient education and dedicated informed consent, a uniform intraoperative strategy (including routine intraoperative neuromonitoring, use of advanced energy-based hemostatic devices, omission of drains, topical hemostatic agents, TIVA-based anesthesia, and systematic parathyroid-protection strategies), and a protocolized postoperative pathway with early PTH measurement, algorithm-driven calcium and vitamin D supplementation, structured surveillance during the first 5–6 postoperative hours, early mobilization, early oral intake, and predefined discharge criteria embedded in a written pathway (Table 5). In addition, the pathway includes a dedicated discharge and follow-up domain, consisting of a comprehensive, digitally generated discharge document summarizing the procedure, postoperative course, and therapies; a structured endocrinology report on thyroid hormone replacement and biochemical monitoring; a predefined schedule of follow-up visits (typically starting at 1 week); and written educational materials (including a 20-question FAQ document) and direct contact details for urgent and non-urgent post-discharge queries.

For the purposes of this economic model, perioperative management was assumed to be clinically equivalent between strategies in terms of intrinsic complication risk, and the comparison was restricted to the organizational dimension of care (timing and setting of postoperative monitoring and discharge) to isolate the impact of early discharge within the Italian DRG framework.

### Model structure and time horizon

A decision-analytic decision tree was constructed to simulate the perioperative course of adult patients undergoing hemithyroidectomy or total thyroidectomy and to capture short-term clinical events and costs over a 30-day horizon (Supplementary Table 4). For each procedure, separate but structurally identical models compared ERAS and DRG pathways, including uneventful recovery, transient hypocalcemia, other early complications (e.g. neck hematoma), emergency department visits, and readmissions. Given the short time horizon, no discounting was applied [18].

### Clinical inputs and safety assumptions

Probabilities of postoperative hypocalcemia, neck hematoma, vocal cord palsy, wound complications, and 30-day readmissions were derived from institutional data and contemporary literature on short-stay thyroidectomy (Supplementary Table 5) [4–13, 19–26]. All patients were assumed to undergo early postoperative PTH testing, with protocolized calcium and vitamin D supplementation when indicated [27, 28], and routine intraoperative neuromonitoring [29–31]. ERAS was assumed not to alter the intrinsic risk of complications but to influence their timing and setting of detection (inpatient vs. outpatient) [32].

### Costing and reimbursement scenarios

Costs were estimated in euros (2025 values) using a micro-costing approach combined with DRG-based reimbursement rules for the SSN [17]. Direct medical costs included ward bed-days, laboratory tests, pharmacologic treatments, diagnostic procedures, and emergency department visits (Supplementary Table 6). Base-case analyses incorporated DRG penalties for length of stay < 2 days, while scenario analyses explored alternative reimbursement settings, including university hospital tariffs, oncology extra-budget activity, tariff indexation, and neutral or private-like reimbursement without early-discharge penalties [15, 16].

### Outcomes and analysis

Primary outcomes included length of stay, direct hospital costs, and contribution margin. Incremental cost-effectiveness was expressed as cost per hospital day avoided; exploratory analyses also considered cost per QALY gained. Expected costs and outcomes were calculated by folding back the decision tree and comparing ERAS and DRG strategies.

### Statistical framework and interpretation of model-based statistics

The decision tree underlying this analysis is deterministic, with parametric uncertainty represented around selected inputs. All p-values and confidence intervals reported in the tables are model-based and are not derived from patient-level hypothesis testing. For length of stay and variable costs, confidence intervals were estimated from empirical distributions (means and standard deviations) observed in our institutional data. For constructed quantities (e.g., number of ward medication administrations and routine laboratory panels), confidence intervals and p-values were derived under the explicit proportionality assumption to length of stay and should be interpreted illustratively rather than inferentially. Parameter uncertainty is further explored through one-way deterministic sensitivity analysis and, for exploratory QALY estimates, through probabilistic sensitivity analysis, in line with established recommendations for decision-analytic models in health-economic evaluation.

### Sensitivity analyses and ethics

Deterministic one-way sensitivity analyses tested the robustness of results to variations in key clinical, cost, and reimbursement parameters, with probabilistic sensitivity analysis performed for exploratory QALY estimates [33, 34]. The study used de-identified aggregate data and published sources and did not require formal ethical approval under national regulations.

## Results

### Modeled cohort and baseline assumptions

The model simulated an annual cohort of 1,000 adult patients undergoing thyroidectomy in a tertiary Italian public hospital operating within the SSN DRG framework, including 400 hemithyroidectomies (40%) and 600 total thyroidectomies (60%). Baseline probabilities of postoperative hypocalcemia, neck hematoma, recurrent laryngeal nerve palsy, wound complications, emergency department (ED) visits, and 30-day readmissions were assumed to be identical between ERAS and conventional DRG-driven pathways, reflecting that organizational models affect timing and setting of care rather than intrinsic surgical risk. Across all simulations, ERAS did not increase major complications or 30-day readmissions for either procedure (all *p* > 0.20; Supplementary Table 7).

### Hemithyroidectomy

In the DRG-driven pathway, mean postoperative length of stay (LOS) was 2.6 ± 0.7 days. Under ERAS, planned same-day discharge after a 6-hour observation period resulted in a mean LOS of 0.33 ± 0.18 days, corresponding to a reduction of 2.27 days per patient (*p* < 0.001). At the cohort level, this freed 908 bed-days per year (*p* < 0.001). Unplanned ED contacts increased modestly (2.5% vs. 5.0%; *p* = 0.041) but without an increase in 30-day readmissions (Table 1).

**Table 1:** Perioperative outcomes for hemithyroidectomy under standard SSN hospitalization versus the ERAS day-surgery pathway, including ward medication and laboratory resource use

Outcome	SSN model	ERAS pathway	Effect (ERAS vs. SSN)	95% CI / *p* value*
Mean postoperative LOS (days)	2.6 ± 0.7 days	0.33 ± 0.18 days	− 2.27 days; − 87.3% hospitalization time	95% CI, 2.18–2.36; *p* < 0.001
Discharge plan	≥ 2 days to preserve DRG tariff	Planned discharge after 6-hour observation	Shift from DRG-driven to protocol-driven LOS	Not applicable
Yearly ward bed-days (400 cases)	1,040 bed-days	132 bed-days	− 908 bed-days freed per year	95% CI, 872–944; *p* < 0.001
Ward medication administrations/patient†	8.0 ± 2.0	1.1 ± 0.4	− 6.9 administrations ( ≈ − 86.3% vs. SSN)	
Ward medication cost/patient (€)	€80 ± 20	€11 ± 5	− €69 per patient ( ≈ − 86.3% vs. SSN)	
Routine lab panels/patient	2.0 ± 0.5	0.3 ± 0.2	− 1.7 panels per patient ( ≈ − 85.0% vs. SSN)	
Lab test cost/patient (€)	€40 ± 10	€6 ± 4	− €34 per patient ( ≈ − 85.0% vs. SSN)	
Total ward med + lab cost/patient (€)	€120 ± 25	€17 ± 7	− €103 per patient ( ≈ − 85–87% vs. SSN)	
Annual ward med + lab cost (400 cases)	€48,000	€6,800	− €41,200 per year	
Patients with ≥ 1 unplanned ED visit	2.5%	5.0%	Increased ED contacts	*p* = 0.041
30-day readmissions	No increase vs. baseline SSN practice reported	No increase despite higher ED contacts	No difference in 30-day readmissions	*p* = NS (no increase observed; CI)

Continuous variables are expressed as mean ± standard deviation; categorical variables are expressed as percentages. Ward medication and laboratory data (administrations per patient, number of lab panels, and related costs) are constructed to be proportional to the observed difference in postoperative LOS and are intended for illustrative health-economic modeling; formal p-values are not reported for these model-derived variables, as conventional hypothesis testing is not applicable to quantities constructed under proportionality assumptions; their uncertainty is explored through sensitivity analysis. Yearly bed-days and annual cost estimates refer to a cohort of 400 hemithyroidectomies. For LOS and yearly ward bed-days, 95% confidence intervals and p values refer to the comparison between SSN and ERAS models as reported in the main analysis

LOS, length of stay; SSN, Servizio Sanitario Nazionale; ERAS, Enhanced Recovery After Surgery; DRG, Diagnosis-Related Group; ED, emergency department; POD, postoperative day; NS, not significant

Mean variable hospital cost decreased from €1,630 ± €417 to €298 ± €147 per case (–€1,332; *p* < 0.001). Although ERAS triggered a 30% DRG tariff reduction for LOS < 2 days (€2,765 vs. €3,950), the contribution margin increased from €2,320 to €2,467 per case (+€147; *p* < 0.001), yielding approximately €58,800 additional annual margin while freeing substantial ward capacity (Table 2).

**Table 2:** Modeled Per-Patient Variable Costs and Provider Margin for Hemithyroidectomy under SSN and ERAS Pathways

Parameter	SSN (Standard Care)	ERAS (Enhanced Recovery)	Absolute Difference	95% CI	*p*-value
Variable hospital cost per case (€)	1,630 ± 417	298 ± 147	− 1,332	− 1,420 to − 1,244	< 0.001
Relative change in variable cost	—	− 81.7%	—	—	—
DRG tariff (DRG 290; hemithyroidectomy)	3,950	2,765*	—	—	—
Financial margin per case (€)	2,320	2,467	+ 147	+ 71 to + 223	< 0.001
Margin rate (financial margin / tariff)	58.7%	89.2%	+ 30.5%	—	—
Annual surgical volume (cases/year)	400	400	—	—	—
Total annual margin (€)	928,000	986,800	+ 58,800	—	—
Annual bed-days occupied / saved	~ 1,100	~ 200	− 900 bed-days	—	—

*A 30% tariff reduction applies for stays of less than 2 days (per SSN rule)

### Total thyroidectomy

For total thyroidectomy, mean LOS decreased from 3.1 ± 0.9 days in the DRG pathway to 1.16 ± 0.45 days under ERAS (–1.94 days; *p* < 0.001), freeing 1,164 bed-days annually. Rates of symptomatic hypocalcemia, neck hematoma, and 30-day readmissions did not differ significantly between pathways, despite more frequent detection of transient biochemical hypocalcemia under ERAS due to systematic early testing (Supplementary Table 9).

Mean variable cost per case decreased from €2,029 ± €497 to €950 ± €251 (–€1,079; *p* < 0.001). However, the DRG penalty for LOS < 2 days reduced reimbursement from €5,200 to €3,640, leading to a decrease in contribution margin from €3,171 to €2,690 per case (–€481; *p* < 0.001). At an annual volume of 600 procedures, this corresponded to a net loss of approximately €288,600 despite major real-resource savings (Table 3).

**Table 3:** Direct hospital costs and provider margin for total thyroidectomy in the SSN setting (ERAS vs. conventional model)

Metric	SSN model (conventional)	ERAS model	Absolute difference (ERAS – SSN)	Relative change	*p* value
Mean variable hospital cost per case, €	2,029 ± 497	950 ± 251	− 1,079 (95% CI − 1,174 to − 984)	− 53.2%	< 0.001
Main drivers of variable costs	Ward bed-days; hotel and nursing costs	Fewer ward bed-days; lower hotel and nursing costs; slightly higher lab tests and ED contacts (+€31 per patient)	–	–	–
DRG tariff per total thyroidectomy (DRG 290), €	5,200	3,640*	− 1,560	− 30.0%	–
Financial margin per case, € (tariff – variable cost)	3,171	2,690	− 481 (95% CI − 557 to − 405)	− 15.2%	< 0.001
Annual contribution margin (600 cases), €	1,902,600	1,614,000	− 288,600	–	–
Annual ward bed-days used (600 cases)	1,860	696	− 1,164	− 62.6%	–

*Tariff reduced by 30% when LOS < 2 days under current SSN DRG rules

### Overall cost-effectiveness and scenario analyses

Across the combined cohort, ERAS reduced mean LOS from 2.95 to 0.86 days (− 2.09 days; *p* < 0.001) and mean variable hospital cost by €1,190 per patient (*p* < 0.001), corresponding to a saving of €569 per hospital day avoided (Table 4). When SSN DRG penalties were applied, these efficiency gains translated into a small net reduction in overall hospital margin, driven entirely by total thyroidectomy.

**Table 4:** Overall cost-effectiveness of ERAS in the SSN public hospital

Outcome / metric	Standard care (per patient)	ERAS pathway (per patient)	Incremental effect (ERAS – standard)	95% CI for increment	*p* value	Interpretation
Mean length of stay, days	2.95	0.86	− 2.09 days	− 2.20 to − 1.98	< 0.001	Substantial reduction in LOS, ~ 2 days fewer per patient
Mean variable hospital cost, €	–	–	− 1,190	− 1,260 to − 1,120	< 0.001	ERAS is **cost-saving** in real provider costs
Incremental cost-effectiveness ratio (ICER)	–	–	− 569 €/hospital day avoided	–	–	Each hospital day avoided saves ~€569 in real resources
Per-patient hospital financial margin, €*	Reference	–	− 229	–	–	Slight reduction in hospital margin due to SSN DRG tariff cuts (TT only)
Net budget impact at hospital level (1,000 pts/year)	–	–	Negative	–	–	Despite efficiency gains, overall hospital budget impact is modestly negative

*Margin calculated after SSN DRG tariffs and penalties; reduction driven entirely by tariff cuts on total thyroidectomy

Scenario analyses showed that modest DRG adjustments (5% university/emergency department uplift or 1% annual tariff indexation) attenuated but did not eliminate this misalignment (Supplementary Tables 9, 10). In contrast, under neutral or private-like reimbursement without early-discharge penalties, ERAS was strongly margin-enhancing, generating approximately €1.2 million in additional annual contribution margin and freeing over 2,000 bed-days per 1,000 procedures (Supplementary Table 11).

## Discussion

This Italian model-based economic evaluation shows that ERAS-style thyroidectomy, when implemented in a high-volume, technology-supported endocrine surgery unit, can substantially reduce hospital resource use and actual provider costs without compromising safety, yet remains structurally penalized by current SSN DRG rules that mandate a minimum two-day postoperative stay for full reimbursement.

### Principal findings and economic implications

The decision-analytic model, calibrated on 1,000 annual thyroidectomies in a tertiary SSN hospital, demonstrates that ERAS reorganizes care around structured short observation after hemithyroidectomy (approximately 6 h) and routine next-day discharge after total thyroidectomy, without increasing major complications or 30-day readmissions compared with DRG-driven conventional care (Supplementary Table 7). From a provider-cost perspective, ERAS was dominant, reducing mean length of stay from 2.95 to 0.86 days per patient and lowering variable hospital costs by approximately €1,190 per case, corresponding to a saving of about €569 per hospital day avoided (Table 4). These findings indicate a clear gain in technical efficiency, with equivalent clinical outcomes achieved using substantially fewer inpatient resources [4–13].

It is important to emphasize that all findings arise from a decision-analytic model calibrated on Italian DRG tariffs and local organizational parameters, and are therefore intrinsically dependent on these context-specific assumptions.

However, when current DRG rules were applied—particularly the 30% tariff reduction for stays under two days—these microeconomic gains translated into a paradoxical reduction in aggregate contribution margin for public hospitals, driven almost entirely by penalized total thyroidectomy cases (Table 3). This highlights a fundamental tension between microeconomic efficiency (minimizing cost per case) and the SSN revenue structure, in which reimbursement remains closely tied to bed occupancy rather than value generation [19–26].

Hospital margins reported here do not represent observed accounting data, but projections under a predefined set of clinical pathways, ward-cost allocations, and reimbursement rules, which should be validated in empirical multi-center costing studies.

Overall, the unit costs of medications, laboratory tests, and routine ward activities are essentially identical for patients managed under ERAS and under the conventional DRG-driven pathway; what truly changes is the number of inpatient days. Consequently, the main driver of cost differences between strategies is the reduction in length of stay, rather than any change in the intensity or price of individual items. In this sense, the model captures a simple but important message: when tariffs and accounting rules are held constant, fewer hospital days translate into lower variable costs for the provider, while the intrinsic safety profile of ERAS thyroidectomy — documented in our institutional experience and in the contemporary literature — remains preserved.

### When efficiency is penalized: structural misalignment in the SSN

Within the SSN DRG framework, any thyroidectomy with a length of stay below two days faces reimbursement cuts regardless of clinical outcomes. In hemithyroidectomy, ERAS reduced mean LOS from 2.6 to 0.33 days and variable costs from €1,630 to €298 per patient, resulting in a modest increase in contribution margin despite DRG penalties and the release of more than 900 bed-days annually (Tables 1 and 2). In contrast, for total thyroidectomy, ERAS reduced LOS from 3.1 to 1.16 days and variable costs from €2,029 to €950 per case, but the associated DRG reduction led to a marked decrease in margin and an annual loss of approximately €288,600 despite freeing over 1,100 bed-days (Table 3).

From a health economics perspective, these unused bed-days represent opportunity costs: capacity that could be reallocated to higher-acuity oncologic or emergency cases is effectively discouraged by a reimbursement system that continues to reward longer stays [19]. Scenario analyses confirmed that modest tariff adjustments, such as university/emergency department uplifts or routine indexation, attenuate but do not resolve this misalignment (Supplementary Tables 9, 10). Only under neutral or private-like reimbursement scenarios—without early-discharge penalties—did ERAS fully translate its intrinsic efficiency into improved hospital margins and capacity release (Supplementary Table 11), consistent with prior reports [4–13, 19–26].

### Safety and feasibility in high-volume settings

In this analysis, ERAS was assumed not to alter the intrinsic risk of postoperative complications, but rather the timing and setting of their detection. Safety relies on strict patient selection and the infrastructure typical of high-volume endocrine surgery units, including standardized open techniques, routine intraoperative neuromonitoring, early postoperative PTH testing with protocolized calcium–vitamin D supplementation, and predefined discharge criteria [4–13, 19–26]. Although ERAS was associated with a modest increase in unplanned emergency department contacts (Table 1), these were generally brief evaluations that did not translate into higher readmission or reintervention rates, supporting the clinical feasibility of early discharge in appropriately selected patients [20, 21].

### ERAS versus short-stay surgery: a conceptual distinction

An important conceptual distinction from our experience is that ERAS should not be equated with “short-stay” surgery. In thyroidectomy, a short stay can be achieved within a conventional care model simply by discharging patients earlier, without significant changes to perioperative processes. In contrast, ERAS refers to a structured, standardized pathway that includes preoperative counseling and consent, intraoperative technology-supported nerve and parathyroid management, protocolized postoperative monitoring, early PTH-guided supplementation, mobilization, and explicit discharge criteria. This is complemented by a dedicated discharge and follow-up domain, which includes comprehensive digital discharge documentation, structured endocrinological planning, scheduled early outpatient review, and patient-centered educational and communication tools (Table 5).

**Table 5:** Structured pre-, intra-, postoperative, and discharge/follow-up components of our ERAS thyroidectomy pathway, highlighting that early discharge is supported by a comprehensive protocol rather than isolated shortening of length of stay

Phase	ERAS component	Description
Preoperative	Structured patient information and education	Outpatient counseling with standardized explanation of procedure, early discharge plan, expected recovery, and warning symptoms.
Preoperative	Dedicated procedure-specific informed consent	Written consent explicitly addressing the ERAS pathway and same-day/overnight discharge.
Preoperative	Educational materials	Two written leaflets: (1) “20 most frequently asked questions about thyroidectomy”; (2) detailed description of admission, surgery, and postoperative course, delivered at clinic visit or pre-admission.
Preoperative	Standardized preoperative assessment	Uniform protocol for clinical evaluation, laboratory tests, and anesthetic assessment.
Intraoperative	Intraoperative neuromonitoring (IONM)	Routine RLN monitoring to standardize nerve identification and management.
Intraoperative	Advanced energy-based and hemostatic devices	Systematic use of energy devices and topical hemostatic agents to minimize bleeding and operative time.
Intraoperative	Parathyroid-protection strategies	Use of autofluorescence and targeted techniques to optimize parathyroid identification and preservation.
Intraoperative	Omission of drains	No cervical drains in standard cases, within a protocol of meticulous hemostasis.
Intraoperative	Standardized TIVA anesthesia	Total intravenous anesthesia according to a predefined protocol.
Postoperative	Early PTH measurement	Systematic PTH testing in the early postoperative period.
Postoperative	Protocol-driven calcium/vitamin D supplementation	Algorithm-based supplementation guided by PTH and calcium thresholds.
Postoperative	Structured early postoperative surveillance	First 5–6 postoperative hours managed with standardized nursing and medical checklists, including neck assessment and pain control.
Postoperative	Early mobilization and oral intake	Ambulation and resumption of oral intake within hours after surgery.
Postoperative	Explicit, written discharge criteria	Predefined criteria for same-day (hemithyroidectomy) or next-day (total thyroidectomy) discharge embedded in the ERAS pathway.
Discharge and follow-up	Comprehensive digital discharge document	Electronic discharge letter including plain-language description of the procedure, postoperative course, prescribed therapies, and individualized recommendations for daily activities and warning signs.
Discharge and follow-up	Structured endocrinology consultation and report	Scheduled postoperative endocrinology visit to define and tailor thyroid hormone replacement, with a written report summarizing indications, targets, and follow-up plan.
Discharge and follow-up	Predefined follow-up appointments	Appointment sheet specifying early outpatient review (typically at 1 week), subsequent visits, and planned laboratory/imaging studies.
Discharge and follow-up	Dedicated contact channels	Provision of a telephone number for urgent issues and a dedicated e-mail address for non-urgent post-discharge questions.
Discharge and follow-up	Post-discharge FAQ leaflet	Additional leaflet with 20 frequently asked questions and answers focused on home management, expected recovery, and criteria for seeking urgent care.

In our unit, early discharge is a downstream consequence of this integrated ERAS protocol, not its defining feature; the pathway is designed to make same-day and next-day discharge safe and reproducible. This distinction is important when interpreting economic evaluations and designing policy incentives: reimbursement systems that reward only reduced length of stay, without recognizing the additional organizational and technological investments required for true ERAS implementation, risk equating simple short-stay models with fully developed ERAS pathways and may inadvertently discourage comprehensive, standardized perioperative care, including structured post-discharge support and endocrine follow-up.

### Strengths and limitations

This study is the first Italian hospital-perspective economic evaluation directly comparing ERAS-based thyroidectomy with conventional SSN DRG-driven hospitalization using detailed micro-costing and realistic reimbursement scenarios [23]. By modeling hemithyroidectomy and total thyroidectomy separately and explicitly capturing short-term postoperative events over a 30-day horizon, the analysis focuses on organizational efficiency rather than crude length-of-stay comparisons.

Limitations include the restriction to adult, low-risk open thyroidectomy cases and the reliance on model assumptions regarding event probabilities and local cost structures. As a model-based analysis, our estimates are sensitive to assumptions regarding unit costs, DRG penalties, and the allocation of fixed versus variable ward costs, and their transferability to hospitals operating under different cost structures or funding schemes is necessarily limited. Social and geographic constraints may limit eligibility for same-day or next-day discharge, and results may differ in low-volume or resource-limited settings. In addition, broader societal costs and long-term outcomes were not considered [25, 26]. Future research should include prospective, multi-center micro-costing studies comparing ERAS-based and conventional thyroidectomy pathways, in order to confirm and refine the hypotheses generated by our model.

### Toward alignment of incentives

Overall, these findings suggest that barriers to ERAS adoption in Italian public thyroid surgery are primarily economic rather than clinical. Revising DRG tariffs to avoid penalizing safe early discharge—through dedicated day-surgery hemithyroidectomy and 24-hour total thyroidectomy pathways—could allow the SSN to capture the real resource savings documented here while preserving patient safety. Accordingly, the policy implications discussed in this paper should be regarded as hypothesis-generating and as possible directions for future tariff redesign — such as dedicated DRGs for day-surgery hemithyroidectomy and 24-hour total thyroidectomy — rather than as prescriptive recommendations. More broadly, aligning reimbursement with evidence-based organizational efficiency may help reduce unwarranted variation between public and private providers and support sustainable, high-quality endocrine surgery within the SSN.

## Supplementary Information

Below is the link to the electronic supplementary material.

Supplementary Material 1

## Data Availability

All data used in this study are either derived from published sources or generated within the decision-analytic model. Additional details on model structure, assumptions, and input parameters are provided in the Supplementary Materials. Further information is available from the corresponding author upon reasonable request.
