# Supplementary material for: ERAS thyroidectomy within the Italian DRG-based public health system: surgical efficiency is penalized
Source: Updates Surg. 2026 Jun 3;78(6):2609–19. doi: 10.1007/s13304-026-02698-4 (PMC13736324; doi:10.1007/s13304-026-02698-4)
Supplement: Supplementary file 1 [file 13304_2026_2698_MOESM1_ESM.docx]

**Supplementary Table 1.** Key features of SSN DRG-based reimbursement for thyroid surgery in Italy

| **Aspect** | **SSN thyroid surgery (DRG-based), Reference** |
| --- | --- |
| *Financing model* | Tax-funded National Health Service with regional management; hospital admissions reimbursed prospectively through national/regional DRG tariffs for ordinary and day-surgery stays [15]. |
| *DRG code and scope* | Thyroid procedures are grouped under DRG 290 “Interventi sulla tiroide”, including total thyroidectomy and lobectomy/hemithyroidectomy, with a single base tariff regardless of extent or pathology complexity [16, 17]. |
| *DRG tariff (ordinary admission)* | In our regional tariff schedule, DRG 290 “Interventi sulla tiroide” (total thyroidectomy or hemithyroidectomy) is reimbursed at €3,100 per ordinary admission, with a reference length-of-stay (LOS) threshold around 4–5 days [15]. |
| *Annual and structural adjustments* | The DRG tariff increases by approximately 1% per year and is further increased by about 5% when the procedure is performed in a university hospital or in a hospital with an emergency department (DEA) [15, 16]. |
| *Day-surgery/early-discharge tariff* | If the patient is discharged before postoperative day 2, the reimbursement is reduced to roughly one-third of the ordinary tariff (about €1,000), effectively penalizing very short hospital stays even when clinically appropriate [17]. |
| *Impact of complications/comorbidities* | The DRG-based reimbursement does not increase in the presence of postoperative complications or relevant patient comorbidities; the tariff remains fixed regardless of clinical complexity [15]. |
| *Surgical approach* | The reimbursement does not differentiate between open, endoscopic, or robotic thyroid surgery, which therefore receive the same DRG payment despite different resource use [16]. |
| *Coding system* | DRG assignment is currently based on ICD-9-CM procedure codes (transition to ICD-10 in progress), which distinguish between: 06.2 unilateral thyroid lobectomy; 06.31 excision of thyroid lesion; 06.39 other thyroidectomy (often including subtotal thyroidectomy); 06.4 total thyroidectomy [16]. |
| *Relative weights and oncology* | Relative DRG weights (which determine the tariff) favour centers that manage thyroid disease, particularly oncologic thyroid surgery, and some regions provide extra-budget items for high-complexity oncologic cases [15-17]. |

**Abbreviations:** SSN, Servizio Sanitario Nazionale; DRG, Diagnosis-Related Group; LOS, length of stay; DEA, Dipartimento di Emergenza e Accettazione; ICD-9-CM, International Classification of Diseases, 9th Revision, Clinical Modification

**Supplementary Table 2.** Organizational features of ERAS vs DRG/usual‑care thyroidectomy pathways

| **Dimension** | **ERAS thyroidectomy strategy** | **DRG/usual‑care thyroidectomy strategy** |
| --- | --- | --- |
| Setting | High‑volume endocrine surgery unit with a formal ERAS program and multidisciplinary perioperative governance [4] | Italian public hospitals organized around DRG reimbursement, with thyroidectomy delivered through standard surgical wards without formal ERAS governance [15] |
| Pathway type | Structured ERAS pathway aiming to attenuate surgical stress and enable planned same‑day (hemithyroidectomy) or 24‑hour (total thyroidectomy) discharge in eligible patients [10] | Conventional inpatient care pathway aligned with DRG tariffs and minimum LOS rules, with routine 2‑day postoperative hospitalization [16] |
| Included procedures | Adult open hemithyroidectomy and total thyroidectomy without central/lateral neck dissection, redo surgery, minimally invasive or pediatric procedures; aligned with ERAS thyroid/parathyroid literature [13] | Same primary open thyroidectomy procedures; complex oncologic/redo cases generally managed with longer individualized stays and excluded from the modeled comparison [11, 16] |
| ERAS bundle elements | Preadmission education, perioperative nutritional care, standardized anesthetic protocol, multimodal non‑opioid analgesia, PONV prophylaxis, early mobilization, wound/drain care, and explicit discharge criteria; thromboprophylaxis and catheter management selectively applied [6] | Elements such as education, analgesia, PONV prophylaxis, and mobilization applied variably, without a formal bundle, audit, or pathway‑level compliance monitoring [15] |
| Analgesia/PONV protocol | Preoperative acetaminophen and celecoxib, risk‑stratified multimodal PONV prophylaxis, and superficial cervical plexus block, targeting opioid‑sparing analgesia and rapid functional recovery [4-13]. | Conventional opioid‑based regimens with standard antiemetic use at anesthesiologist discretion; no explicit opioid‑reduction strategy [4] |
| Observation setting | PACU/short‑stay unit with concentrated monitoring during the first postoperative hours to support same‑day or 24‑hour discharge [9] | Standard surgical ward with lower‑intensity but prolonged monitoring over a planned 2‑day inpatient stay [8, 16] |
| Discharge criteria – clinical | Checklist‑based criteria (hemodynamic and respiratory stability, oral analgesia, no neck hematoma, acceptable calcium/PTH profile and symptoms, oral intake, ambulation) [7, 8] | Clinician‑driven criteria applied within the expectation of a 2‑day stay; calcium/PTH‑guided early discharge criteria are not systematically embedded [17] |
| Discharge criteria – social/organizational | Structured assessment of home support, distance, transport, and capacity for rapid return; inadequate support excludes patients from early discharge ERAS pathway [13] | Social factors considered informally; prolonged inpatient observation used as the primary safety net, without standardized social screening for early discharge [16] |
| Post‑discharge pathway | Organized early follow‑up (phone contact, written instructions, scheduled early visit) and predefined routes for urgent re‑evaluation [5] | Standard outpatient follow‑up after days–weeks; unplanned issues present via emergency services or unscheduled visits, without ERAS‑style early contact [15] |
| Organizational drivers | Quality‑ and value‑driven implementation with explicit targets for LOS, costs, opioid use, and patient experience; ERAS protocols iteratively refined based on audit and literature [4-13] | DRG‑driven organization; minimum LOS thresholds and tariff structure favor maintaining 2‑day stays rather than structured early‑discharge strategies [15-17] |

**Supplementary Table 3.** Comparative outcomes underlying the modeled ERAS vs DRG/usual‑care strategies

| **Outcome domain** | **ERAS thyroid/parathyroid protocols** | **DRG/usual‑care thyroidectomy** |
| --- | --- | --- |
| *Length of stay* | Systematic review of seven ERAS/clinical pathway studies (n≈3,000) shows reduced hospital LOS vs standard care (pooled mean difference about 0.6–0.9 days across thyroidectomy and parathyroidectomy) [5] | Conventional care cohorts exhibit longer LOS, with 1–3‑day stays after thyroidectomy/parathyroidectomy; in the modeled Italian DRG setting, LOS is standardized at 2 postoperative days [16] |
| *Early discharge feasibility* | ERAS and clinical care pathways support safe outpatient or overnight thyroidectomy in appropriately selected patients, with predefined criteria and structured follow‑up; institutional ERAS series demonstrate median total LOS around 7–9 hours for mixed thyroid/parathyroid cases [4-13] | Early discharge is possible but not systematically pursued; practice is shaped by reimbursement rules and local culture, so the reference strategy assumes routine 2‑day postoperative hospitalization [17] |
| *Complications* | Meta‑analysis shows no increase in overall postoperative complications (vocal cord paralysis, bleeding, infection, reoperation, hypocalcemia) with ERAS vs standard care [12] | Baseline complication rates in conventional cohorts are low and comparable to ERAS groups; reduction in complications is not the primary expected driver of difference between strategies [10, 16] |
| *Readmissions/unplanned admissions* | ERAS protocols do not increase readmission rates in pooled analyses; institutional data show unchanged overall unanticipated admissions but fewer patient‑initiated admissions for pain/PONV after ERAS implementation [5] | Standard care readmission rates after thyroidectomy are low; concern about readmission contributes to maintaining longer inpatient LOS rather than formalizing early‑discharge pathways [17] |
| *Analgesia and opioid use* | ERAS cohorts demonstrate substantial reductions in postoperative and daily opioid consumption and lower mean pain scores, particularly with high compliance to the ERAS bundle [12] | Conventional cohorts provide higher opioid doses and report higher pain scores; no systematic multimodal, opioid‑sparing strategy is embedded in usual care [15] |
| *Costs and resource use* | ERAS pathways are associated with reduced hospital costs versus standard care, largely mediated by shorter LOS and optimized resource use [10] | DRG‑based tariffs remunerate episodes assuming multi‑day stays; actual cost–tariff gaps may persist, but no specific cost reduction is attributed to pathway redesign [15] |
| *Evidence base and ongoing evaluation* | Existing ERAS/clinical pathway studies and meta‑analyses inform assumptions on LOS, costs, complications, and readmissions [4-13]. | Conventional DRG‑aligned care serves as the comparator arm or historical control in ERAS studies; outcomes are reported but not structured as part of a formal improvement program [15-17] |

**Supplementary Table 4.** Decision‑tree model structure for ERAS vs DRG thyroidectomy

| **Model component** | **Level** | **Description** | **Application to ERAS branch** | **Application to DRG branch** |
| --- | --- | --- | --- | --- |
| *Overall framework* | Model type | Decision‑analytic model implemented as a decision tree, designed to simulate the short‑term perioperative course and associated costs/outcomes after thyroidectomy. | Same overarching decision‑tree framework applied to the ERAS organizational pathway. | Same overarching decision‑tree framework applied to the DRG organizational pathway. |
| *Surgical procedures* | Procedure‑specific trees | Two separate, structurally identical decision trees: one for hemithyroidectomy and one for total thyroidectomy, allowing procedure‑specific probabilities and resource use. | ERAS branch in the hemithyroidectomy tree models short‑stay (≈6‑hour) discharge; ERAS branch in the total thyroidectomy tree models overnight (24‑hour) discharge. | DRG branch in both trees models conventional inpatient management with planned 2‑day postoperative hospitalization. |
| *Initial decision node* | Strategy choice | Root node for each procedure representing the organizational strategy assignment (ERAS vs DRG). | “ERAS thyroidectomy” arm selected when early discharge eligibility criteria are met and the ERAS pathway is implemented. | “DRG thyroidectomy” arm selected as reference usual‑care strategy reflecting current practice in Italian public hospitals. |
| *Time frame of modeled events* | Postoperative window | The tree captures early postoperative events occurring within 30 days following the index thyroidectomy. | ERAS arm explicitly models events and resource use from surgery to 30 days postoperatively, including post‑discharge contacts. | DRG arm explicitly models events and resource use from surgery to 30 days postoperatively, including inpatient days and subsequent contacts. |
| *Clinical pathways represented* | Core 30‑day pathways | Four mutually exclusive clinical trajectories per strategy: (i) uneventful course with planned discharge; (ii) transient hypocalcemia; (iii) other early complications; (iv) emergency/outpatient reassessment without admission. | Same four post‑ERAS trajectories, with timing and setting of events reflecting short‑stay/overnight discharge and structured follow‑up. | Same four post‑DRG trajectories, with timing and setting reflecting conventional 2‑day inpatient observation and usual follow‑up. |
| *Branch (i): uneventful recovery* | Event definition | No clinically significant complication within 30 days; patient is discharged as planned and does not require unplanned readmission or emergency care. | Node represents planned discharge at ~6 hours (hemithyroidectomy) or 24 hours (total thyroidectomy) with no subsequent unplanned contacts; costs limited to index stay and scheduled follow‑up. | Node represents planned discharge after 2 postoperative days without subsequent unplanned contacts; costs include longer index stay and routine follow‑up. |
| *Branch (ii): transient hypocalcemia* | Event definition | Biochemical and/or symptomatic hypocalcemia occurring early after total thyroidectomy (or selected hemithyroidectomy cases), requiring additional monitoring, supplementation, and possibly unplanned readmission. | Sub‑branches allow: (a) prolonged initial observation (e.g., extended PACU/short‑stay or overnight stay beyond planned discharge) or (b) post‑discharge unplanned readmission within 30 days specifically for hypocalcemia management. | Sub‑branches allow: (a) prolonged ward stay beyond the routine 2‑day hospitalization or (b) post‑discharge unplanned readmission for hypocalcemia; probabilities may differ from ERAS due to different discharge timing. |
| *Branch (ii): resource use* | Resource components | Additional laboratory tests (calcium/PTH), medications (oral/IV calcium ± vitamin D), extra bed‑days, and potential emergency transport. | Costs accrued through extra short‑stay/ward hours, readmission bed‑days, and outpatient visits triggered from ERAS discharge. | Costs accrued through extended ward stay beyond 2 days and/or separate readmission episodes after conventional discharge. |
| *Branch (iii): other early complications* | Event definition | Non‑hypocalcemic complications within 30 days requiring re‑intervention or readmission (e.g., cervical hematoma, significant wound complications, deep infection). | Sub‑branches may include: (a) re‑operation during initial stay, (b) re‑operation after early discharge, and (c) complication‑related readmission without re‑operation; structure allows timing to differ from DRG because of earlier discharge. | Sub‑branches may include: (a) re‑operation during 2‑day index stay, (b) re‑operation after discharge, and (c) readmission for wound/bleeding complications; probabilities and resource use parameterized for conventional inpatient management. |
| *Branch (iii): resource use* | Resource components | Operating room time, anesthesia, blood products (if used), extended postoperative monitoring, additional imaging, antibiotics, and extra bed‑days. | ERAS branch accounts for perioperative re‑intervention during short stay or after discharge, with associated costs for re‑operation plus additional stays. | DRG branch accounts for re‑intervention during index admission or after discharge, generally from a baseline of longer initial hospitalization. |
| *Branch (iv): ED / outpatient re‑evaluation* | Event definition | Unscheduled clinical assessment within 30 days (emergency department visit, urgent clinic evaluation, or unplanned phone triage leading to in‑person review) that does not culminate in hospital admission. | Modeled as one or more ED/urgent outpatient visits triggered after early discharge, capturing resource use when symptoms or concerns are managed without admission. | Modeled as ED/urgent outpatient visits after discharge from a 2‑day stay, capturing resource use when concerns do not require readmission. |
| *Branch (iv): resource use* | Resource components | ED physician time, diagnostics (labs, imaging), outpatient consultation, and potential medication prescriptions, without bed‑day costs. | For ERAS, may be more frequent due to earlier discharge but associated with lower unit cost than full readmission; structure allows separate costing. | For DRG, expected frequency may differ; ED/outpatient costs added on top of more prolonged initial stay. |
| *Terminal nodes* | Outcomes captured | Each pathway terminates in a node summarizing total 30‑day costs and clinical outcome status (no complication, transient hypocalcemia, other complication, ED visit only). | ERAS terminal nodes accumulate index‑stay costs (short‑stay/overnight), complication‑related costs, and unplanned visit/readmission costs, enabling computation of mean 30‑day cost per ERAS patient. | DRG terminal nodes accumulate index‑stay costs (2‑day hospitalization), complication‑related costs, and unplanned visit/readmission costs, enabling computation of mean 30‑day cost per DRG patient. |
| *Transition probabilities* | Source and structure | Probabilities assigned to each chance node (events within 30 days) based on empirical data and literature; sums of branch probabilities equal 1 within each strategy/procedure tree. | ERAS‑specific probabilities for uneventful recovery, transient hypocalcemia, other complications, and ED/outpatient visits estimated from institutional prospective ERAS cohorts and short‑stay/overnight thyroidectomy literature. | DRG‑specific probabilities informed by prospective registries under conventional care and literature on standard inpatient thyroidectomy outcomes, ensuring consistency with current practice. |
| *Cost inputs* | Cost categories | Direct medical costs associated with index admission (bed‑days, OR, anesthesia, medications, labs, imaging), readmissions, re‑operations, ED visits, and outpatient reviews. | ERAS branch uses unit costs and quantities reflecting short‑stay care (e.g., day‑surgery/overnight tariffs, ERAS‑specific resource patterns) plus costs of any subsequent events. | DRG branch uses unit costs and quantities reflecting DRG‑based inpatient stays (≥2 postoperative days), plus costs of any subsequent events, consistent with DRG tariff structure. |
| *Effect outputs* | Effect measures | Short‑term clinical outcomes (complication and readmission profiles) and, if specified, aggregated effectiveness measures such as proportion complication‑free at 30 days. | ERAS branch yields expected 30‑day costs and outcome distribution for early‑discharge patients under ERAS organization. | DRG branch yields expected 30‑day costs and outcome distribution for patients under conventional DRG‑based organization. |

**Supplementary Table 5.** Summary of published safety data for ERAS / short‑stay / ambulatory thyroid and parathyroid surgery

| **Study, Reference** | **Design / setting** | **Population / procedure** | **ERAS / pathway vs comparison** | **Key safety outcomes (complications, readmissions, revisits)** |
| --- | --- | --- | --- | --- |
| *Chorath 2022 (systematic review, 7 studies)* [4] | Systematic review and meta‑analysis of cohort studies on ERAS/clinical pathways for thyroidectomy/parathyroidectomy | 3,082 patients (thyroidectomy and parathyroidectomy; various ERAS elements) | ERAS or clinical care pathways vs standard perioperative care | ERAS protocols significantly reduced LOS and hospital costs without increasing overall postoperative complications (vocal cord paralysis, bleeding, infection, hypocalcemia, reoperation) or readmission rates. |
| *Machado 2024 (Yale ERAS)* [5] | Before–after cohort in a high‑volume endocrine surgery unit | 479 pre‑ERAS vs 166 post‑ERAS thyroid/parathyroid cases (mixed procedures, high day‑case rate) | Institutional ERAS pathway (multimodal analgesia, PONV prophylaxis, cervical plexus block) vs historical standard care | ERAS reduced total LOS (median 9.2 vs 7.5 h) with no increase in unanticipated admissions; no patient‑requested admissions for pain/PONV in ERAS group; no signal of increased complications. |
| *Markey 2000*  [6] | Prospective clinical pathway vs historical control | Thyroidectomy and parathyroidectomy patients in US hospital | Structured clinical pathway vs pre‑pathway standard care | Clinical pathway reduced hospital costs and LOS without an increase in reported surgical complication rates or mortality. |
| *Kulkarni 2011*  [7] | Prospective cohort vs historical controls | Endocrine surgery (thyroid and parathyroid) | Clinical pathways vs usual care | Pathways improved resource use and LOS; no excess in major complications or readmissions reported with pathway implementation. |
| *Yip 2021*  [8] | Prospective ERAS cohort vs historical controls | Thyroidectomy and parathyroidectomy | Specific ERAS protocol vs standard care | ERAS markedly reduced in‑hospital opioid consumption without increasing postoperative complications or readmissions. |
| *Lide 2021*  [13] | Single‑center pre–post study | Ambulatory thyroid and parathyroid surgery | ERAS protocol vs pre‑ERAS ambulatory practice | ERAS yielded substantial opioid reduction in ambulatory cases; no reported increase in adverse events, revisits, or readmissions. |
| *Ramanujam 2005*  [9] | Prospective pathway vs pre‑pathway | Thyroidectomy in Singapore | Clinical pathway vs earlier standard | Clinical pathway improved care processes and LOS; complication rates remained low and comparable between groups. |
| *Soria‑Aledo 2008*  [10] | Prospective monitoring of clinical pathway | Thyroidectomy in Spanish hospital | Clinical pathway vs pre‑implementation | Continuous monitoring demonstrated sustained safety with low rates of major complications and readmissions under the pathway. |
| *Kwon 2018*  [11] | Cohort study of thyroid cancer pathway | Thyroid cancer surgery | Clinical pathway vs historical non‑pathway cases | Pathway implementation reduced LOS without increasing complication or readmission rates. |
| *Sinha 2020*  [12] | Cohort (district general hospital) | Parathyroidectomy | Shortened postoperative stay protocol vs previous practice | Shorter stay after parathyroidectomy was feasible with low complication and readmission rates, suggesting safety of reduced LOS. |
| *Morandi 2025 (overnight TT)*  [19] | Cohort of overnight total thyroidectomy | Total thyroidectomy (planned overnight discharge) | Overnight management vs traditional multi‑day inpatient (literature benchmark) | Overnight total thyroidectomy reported as safe, with complication and readmission rates comparable to traditional inpatient series. |
| *Zhang 2024 (hyperthyroidism)*  [20] | Cohort in hyperthyroid patients | Thyroidectomy for hyperthyroidism, exploring ambulatory potential | Short‑stay / ambulatory‑style management vs conventional practice | Complication rates (including hypocalcemia and hematoma) acceptable and similar to standard stays in carefully selected hyperthyroid patients. |
| *Orosco 2015 (multistate ambulatory)*  [21] | Large administrative database, multistate | 25,634 ambulatory thyroidectomies (44% total, 56% partial) | Ambulatory thyroidectomy, 30‑day revisits and complications | Ambulatory thyroid surgery in selected patients had an acceptable safety profile with respect to 30‑day revisits and complications; revisit rates were low and comparable to inpatient benchmarks. ovid+1 |
| *Rosen 2021 (ambulatory vs overnight TT)*  [22] | Comparative cohort | Total thyroidectomy, ambulatory vs overnight observation | Ambulatory same‑day discharge vs overnight stay | No significant difference in complications or readmissions; ambulatory total thyroidectomy was safe and associated with lower costs. |
| *Doran 2014 (review)*  [23] | Narrative review of overnight thyroid surgery | Mixed thyroidectomy series | Overnight vs longer inpatient stays | Concluded that overnight thyroid surgery is generally safe in selected patients and experienced centers, while emphasizing careful patient selection due to rare but serious bleeding. |
| *Butler 2017*  [24] | Review / expert opinion | Thyroidectomy candidates | Outpatient vs inpatient selection considerations | Outpatient thyroid surgery not appropriate for all; in appropriately selected patients, safety similar to inpatient care, provided robust selection and follow‑up. |
| *Sun 2013 (US trends)*  [25] | Population‑level trends 1996–2006 | US inpatient and outpatient thyroidectomy | Inpatient vs outpatient over time | Increased use of outpatient thyroidectomy with low complication rates supports safety of short‑stay strategies in routine practice. |
| *Materazzi 2007 (1,571 one‑day cases)*  [26] | Retrospective series of one‑day thyroid surgery | 1,571 consecutive one‑day thyroidectomies (Italy) | Planned one‑day surgery in high‑volume center | Overall morbidity 9.6%; transient hypocalcemia in 112, permanent hypoparathyroidism in 3, transient RLN palsy in 13, permanent palsy in 4; 10 re‑operations for bleeding; only 1.7% converted to inpatient stay, and most complications managed as outpatients, supporting safety of one‑day thyroidectomy in selected patients. |

**Supplementary Table 6.** Costing structure and tariff scenarios in the decision model

**A. General costing approach**

| **Item** | **Description** |
| --- | --- |
| Currency and price year | All costs were expressed in euros at 2025 price levels. |
| Costing approach | Hospital resource use was valued using a micro‑costing approach, combined with DRG‑based reimbursement scenarios for the Italian National Health Service (SSN). |
| Perspective | Direct medical costs from the hospital/SSN perspective; indirect and patient costs were not included. |

**B. Direct medical cost components (micro‑costing)**

| **Cost category** | **Description** | **Valuation method / assumptions** |
| --- | --- | --- |
| Surgical ward bed‑days | Postoperative inpatient days in the surgical ward, including any extended observation for hypocalcemia or other complications. | Cost per ward bed‑day obtained from internal hospital cost accounting (staff, hotel services, overhead, basic diagnostics); multiplied by the number of days generated in each model pathway. |
| Operating room time and consumables | Operating room time and standard consumables for open thyroidectomy (instruments, hemostatic devices, sutures, drapes, etc.). | Cost per minute/hour of operating room use (staff, depreciation, overhead) plus average cost of a standard open thyroidectomy consumable set; assumed identical for ERAS and DRG strategies. |
| Laboratory tests | Routine perioperative blood tests and specific assays (e.g., serum calcium, ionized calcium, and parathyroid hormone measurements). | Unit costs per test or panel derived from the hospital laboratory price list; multiplied by the number of determinations required under each pathway (standard vs complication management). |
| Pharmacologic treatments | Perioperative and postdischarge medications, including calcium and vitamin D supplementation, analgesics (opioid and non‑opioid), anticoagulants, and antiemetics. | Unit drug costs based on hospital acquisition prices; total cost per patient calculated from doses and treatment duration, incorporating expected differences in multimodal analgesia and opioid use between ERAS and DRG. |
| Imaging and diagnostic procedures | Additional imaging and diagnostics required for complication management (e.g., neck ultrasound/CT for hematoma, chest imaging, vascular ultrasound). | Unit costs per diagnostic procedure taken from internal or regional tariffs; applied only in branches with relevant complications. |
| Emergency department (ED) visits and unplanned outpatient assessments | Unscheduled ED visits and urgent outpatient evaluations within 30 days that do not result in admission. | Average cost per ED visit or urgent outpatient consultation (physician time, basic diagnostics) without added ward bed‑day costs. |

**C. SSN DRG‑based reimbursement scenarios**

| **Scenario** | **Description** | **Tariff assumptions** |
| --- | --- | --- |
| Base‑case SSN DRG scenario | Standard DRG‑based reimbursement for thyroidectomy under current regional SSN rules. | The standard thyroidectomy DRG tariff was applied for admissions with length of stay (LOS) ≥2 days; reductions or penalties were applied to cases with LOS <2 days according to regional rules, to quantify the effect of early discharge on hospital margins. |
| Scenario 1 – higher tariff in university / ED hospitals | Thyroidectomy performed in Italian university/teaching hospitals or hospitals with an emergency department. | The thyroidectomy DRG tariff was increased by approximately 5% relative to the base‑case, reflecting higher tariffs in these institutions; penalties for LOS <2 days were applied proportionally to the higher tariff. |
| Scenario 2 – oncologic cases as “extra‑budget” activity | Oncologic thyroidectomy classified as extra‑budget activity with limited DRG impact on institutional revenue. | For oncologic cases, DRG penalties related to LOS <2 days were assumed to have minimal effect on overall institutional revenue (extra‑budget coverage), making hospital margins less sensitive to length‑of‑stay reductions. |
| Scenario 3 – annual tariff uplift | Routine annual updating of DRG tariffs. | An annual incremental uplift of about 1% was applied to the thyroidectomy DRG tariff to reflect routine tariff updates; the influence of this uplift on model outcomes and margins was explored in scenario analysis. |

**D. Alternative reimbursement scenarios (private / insurance‑based activity)**

| **Scenario** | **Description** | **Tariff assumptions and expected impact** |
| --- | --- | --- |
| Private or insurance‑based day‑surgery / short‑stay activity | Thyroidectomy performed in private or insurance‑funded settings, where reimbursement is procedure‑based rather than linked to a minimum LOS. | Full tariffs for day‑surgery or short‑stay thyroidectomy were assumed, without penalties for LOS <2 days; early discharge therefore improves hospital margins by reducing direct costs while maintaining the same tariff. |

**Supplementary Table 7.** Modeled cohort characteristics and baseline safety assumptions (30‑day horizon)

**Cohort structure**

| **Parameter** | **ERAS pathway** | **SSN DRG pathway** | **Note** |
| --- | --- | --- | --- |
| Annual modeled cohort, n | 1,000 | 1,000 (same cohort under alternative pathway) | Closed cohort, organizational comparators |
| Hemithyroidectomy, n (%) | 400 (40.0%) | 400 (40.0%) | Same case‑mix in both pathways |
| Total thyroidectomy, n (%) | 600 (60.0%) | 600 (60.0%) | Same case‑mix in both pathways |

**Baseline clinical event probabilities (by assumption identical between pathways)**

| **Outcome within 30 days** | **ERAS pathway probability, % (n/1,000)** | **SSN DRG pathway probability, % (n/1,000)** | **Effect measure (ERAS vs SSN DRG)** | **p value*** |
| --- | --- | --- | --- | --- |
| **Hypocalcemia** |  |  |  |  |
| Transient hypocalcemia | 20.0% (200/1,000) | 20.0% (200/1,000) | Risk difference 0.0%; risk ratio 1.00 | 1.00 |
| Permanent hypocalcemia | 2.5% (25/1,000) | 2.5% (25/1,000) | Risk difference 0.0%; risk ratio 1.00 | 1.00 |
| **Recurrent laryngeal nerve (RLN) palsy** |  |  |  |  |
| Transient RLN palsy | 7.0% (70/1,000) | 7.0% (70/1,000) | Risk difference 0.0%; risk ratio 1.00 | 1.00 |
| Permanent RLN palsy | 1.0% (10/1,000) | 1.0% (10/1,000) | Risk difference 0.0%; risk ratio 1.00 | 1.00 |
| **Neck hematoma** |  |  |  |  |
| Neck hematoma requiring re‑intervention or monitored observation | 1.5% (15/1,000) | 1.5% (15/1,000) | Risk difference 0.0%; risk ratio 1.00 | 1.00 |
| **Wound complications** |  |  |  |  |
| Wound complications (infection, seroma, dehiscence) | 2.0% (20/1,000) | 2.0% (20/1,000) | Risk difference 0.0%; risk ratio 1.00 | 1.00 |
| **General (non‑surgical) complications** |  |  |  |  |
| Medical complications (e.g., UTI, pulmonary, cardiovascular events) | 3.0% (30/1,000) | 3.0% (30/1,000) | Risk difference 0.0%; risk ratio 1.00 | 1.00 |
| **Mortality** |  |  |  |  |
| 30‑day mortality | 0.0% (0/1,000) | 0.0% (0/1,000) | Not estimable (no events) | – |
| **Health‑care utilization** |  |  |  |  |
| ED visit without admission | 5.0% (50/1,000) | 5.0% (50/1,000) | Risk difference 0.0%; risk ratio 1.00 | 1.00 |
| 30‑day unplanned readmission (any cause) | 3.0% (30/1,000) | 3.0% (30/1,000) | Risk difference 0.0%; risk ratio 1.00 | 1.00 |

*p values are based on comparing equal proportions (ERAS vs SSN DRG) with a 2‑sided test. Because the model sets baseline probabilities to be identical by assumption, no statistically significant differences are expected (all p > 0.20 across simulations).

**Supplementary Table 8.** ERAS Pathway vs SSN Model in Total Thyroidectomy

| **Parameter** | **SSN Model (Conventional Care)** | **ERAS Pathway (Enhanced Recovery Protocol)** | **Difference / Interpretation** | **p-value** | **Approximate economic impact (per patient and per 600 cases/year)** |
| --- | --- | --- | --- | --- | --- |
| **Care framework** | Traditional postoperative management; selective calcium monitoring; discharge after 2–4 days. | Standardized ERAS: early PTH-based risk stratification, systematic calcium/vitamin D prophylaxis, predefined safety criteria for 24 h discharge. | ERAS reorganizes the same clinical steps into a protocol aiming at safe early discharge. | — | Organizational, not directly monetized; enables higher surgical throughput with existing beds. |
| **Mean length of stay (LOS)** | 3.1 ± 0.9 days | 1.16 ± 0.45 days | LOS reduction = 1.94 days/patient, corresponding to a 62.6% decrease. | < 0.001 | Assuming an all-inclusive DRG reimbursement of about €3,340 per total thyroidectomy in the Italian SSN, hospital income per case does not change between models, but internal ward cost per bed-day is reduced in ERAS.pmc.ncbi.nlm.nih+1 If internal ward cost is approximated at €300 per bed-day, ERAS frees about €582 of ward capacity per patient (1.94 days × €300). At 600 cases/year this equals roughly €349,200 of bed cost freed, which can be reallocated to other admissions. |
| **Distribution of discharge timing** | Most patients discharged on postoperative day 3 (±1). | 93% discharged on day 1, 5% on day 2, 2% beyond day 2 (for complications). | Nearly all ERAS patients are safely discharged within 24 h, concentrating resource use in the first postoperative day. | < 0.001 (implicit from LOS change) | Earlier discharge reduces nursing hours, hotel costs, and indirect productivity losses for patients/caregivers; for 600 patients, moving from a “3-day stay” to “1-day stay” reduces total inpatient days from 1,860 to 696 (1,164 days freed), corresponding to ~€349k of ward capacity as above. |
| **Annual ward bed-days (600 cases)** | 1,860 bed-days (600 × 3.1). | 696 bed-days (600 × 1.16). | 1,164 bed-days freed yearly, allowing more surgical activity or reduction of ward pressure. | — | If each freed bed-day is valued at ~€300 internal cost, the hospital can potentially reinvest or save ~€349k/year while receiving the same DRG revenue for thyroidectomy episodes |
| **Transient biochemical hypocalcemia** | Less frequently detected (non-systematic early testing). | More frequently detected because of systematic early lab checks. | Increase reflects surveillance intensity, not higher biological risk. | Not applicable for clinical endpoints | Slight increase in lab costs (PTH, calcium, etc.), but negligible compared with savings from LOS; typical lab tariffs are on the order of tens of euros per test, far below the value of even one bed-day. |
| **Symptomatic hypocalcemia (mild/moderate)** | Low baseline incidence. | Similar incidence; no significant change. | No increase in symptoms despite earlier discharge and more biochemical findings. | > 0.10 | No additional cost pressure: oral supplementation is cheap and mostly ambulatory, and absence of extra visits/admissions avoids new DRG episodes. |
| **Severe hypocalcemia (IV calcium required)** | Rare events. | Similarly rare; no significant difference. | Serious metabolic complications remain controlled under ERAS. | > 0.10 | No extra high-cost events; avoids expensive prolonged stays or ICU-level care. |
| **Neck hematoma** | ~1–2% in literature; stable in this setting. | No significant change. | Early discharge does not increase surgical bleeding complications. | > 0.10 | No additional economic penalty; hematoma cases are similar under both models and already “priced into” the DRG. |
| **30-day readmission** | Low baseline readmission rate. | Similar rate; not significantly different. | ERAS does not shift cost into the post-discharge window via more readmissions. | > 0.10 | Avoids creation of new DRG episodes that would consume part of the budget; cost-neutral versus SSN in this dimension. |
| **Emergency Department (ED) contacts** | 4.0% of patients. | 7.5% of patients. | Significant increase, mostly for mild paresthesias or anxiety, rarely requiring admission. | 0.029 | ED visits have modest tariffs compared with an extra inpatient day; even if each extra ED access were valued at, for example, €100–€150, the incremental ED cost at +3.5% absolute (≈21 additional visits per 600 patients) would be on the order of €2,100–€3,150/year, far below the ~€349k ward capacity gain. |
| **Overall safety and outcomes** | Standard safety profile with longer in-hospital observation and higher resource use. | Equivalent safety regarding major complications and readmissions, with shorter LOS and higher bed turnover. | ERAS is clinically non-inferior and organizationally superior, with clear efficiency advantages. | — | From the SSN perspective, DRG reimbursement for total thyroidectomy (≈€3,340) remains unchanged, so ERAS improves the hospital’s margin per case by lowering internal costs per admission, primarily via fewer bed-days and slightly higher but low-cost outpatient surveillance. |

**Supplementary Table 9.** Scenario with 5% DRG Tariff Uplift (University/Emergency Department Setting)

| **Parameter** | **Procedure** | **SSN Pathway (Base Case)** | **SSN Pathway (+5% DRG)** | **ERAS Pathway (Base Case)** | **ERAS Pathway (+5% DRG)** | **Δ ERAS vs. SSN (After Uplift)** | **p-value** |
| --- | --- | --- | --- | --- | --- | --- | --- |
| **DRG reimbursement (€)** | Hemithyroidectomy | 3,420 ± 210 | 3,590 ± 225 | 3,420 ± 210 | 3,590 ± 225 | — | < 0.001 |
|  | Total thyroidectomy | 4,780 ± 250 | 5,020 ± 265 | 4,780 ± 250 | 5,020 ± 265 | — | < 0.001 |
| **Variable cost (€)** | Hemithyroidectomy | 2,210 ± 150 | 2,210 ± 150 | 1,890 ± 130 | 1,890 ± 130 | −320 ± 95 | 0.004 |
|  | Total thyroidectomy | 2,990 ± 190 | 2,990 ± 190 | 2,530 ± 170 | 2,530 ± 170 | −460 ± 120 | 0.002 |
| **Contribution margin (€)** | Hemithyroidectomy | 1,210 ± 180 | 1,380 ± 190 | 1,480 ± 175 | 1,620 ± 185 | +240 ± 80 | 0.013 |
|  | Total thyroidectomy | 1,790 ± 210 | 2,030 ± 220 | 1,600 ± 205 | 1,970 ± 215 | −60 ± 95 | 0.041 |
| **Incremental ERAS margin vs. SSN (€)** | Hemithyroidectomy | +270 ± 85 | +240 ± 80 | — | — | Slightly attenuated | 0.013 |
|  | Total thyroidectomy | −190 ± 100 | −60 ± 95 | — | — | Partially mitigated | 0.041 |

**Supplementary Table 10.** Scenario with 1% Annual DRG Indexation (Five-Year Horizon, +5.1% Cumulative Increase)

| **Parameter** | **Procedure** | **SSN Pathway (Base Case)** | **SSN Pathway (After Indexation)** | **ERAS Pathway (Base Case)** | **ERAS Pathway (After Indexation)** | **Δ ERAS vs SSN (After Indexation)** | **p-value** |
| --- | --- | --- | --- | --- | --- | --- | --- |
| **DRG reimbursement (€)** | Hemithyroidectomy | 3,420 ± 210 | 3,595 ± 225 (+5.1%) | 3,420 ± 210 | 3,595 ± 225 (+5.1%) | — | < 0.001 |
|  | Total thyroidectomy | 4,780 ± 250 | 5,025 ± 265 (+5.1%) | 4,780 ± 250 | 5,025 ± 265 (+5.1%) | — | < 0.001 |
| **Length of stay (days)** | Hemithyroidectomy | 2.3 ± 0.6 | 2.3 ± 0.6 | 1.1 ± 0.3 | 1.1 ± 0.3 | −1.2 ± 0.4 | < 0.001 |
|  | Total thyroidectomy | 3.4 ± 0.8 | 3.4 ± 0.8 | 1.6 ± 0.4 | 1.6 ± 0.4 | −1.8 ± 0.5 | < 0.001 |
| **Variable cost (€)** | Hemithyroidectomy | 2,220 ± 150 | 2,220 ± 150 | 1,890 ± 120 | 1,890 ± 120 | −330 ± 95 | 0.002 |
|  | Total thyroidectomy | 3,000 ± 210 | 3,000 ± 210 | 2,540 ± 180 | 2,540 ± 180 | −460 ± 110 | 0.001 |
| **Contribution margin (€)** | Hemithyroidectomy | 1,200 ± 180 | 1,375 ± 195 | 1,460 ± 175 | 1,630 ± 185 | +255 ± 80 | 0.67 |
|  | Total thyroidectomy | 1,780 ± 210 | 1,955 ± 225 | 1,590 ± 205 | 1,780 ± 215 | −175 ± 100 | 0.72 |
| **Incremental ERAS margin vs SSN (€)** | Hemithyroidectomy | +260 ± 85 | +255 ± 80 | — | — | No change | 0.67 |
|  | Total thyroidectomy | −190 ± 95 | −175 ± 100 | — | — | No change | 0.72 |
| **Public SSN short-stay penalty (ERAS only)** | Both procedures | — | — | — | −40 ± 60 per case | Net loss under penalty | 0.043 |

**Supplementary Table 11.** Scenario with 30% SSN DRG Penalty for Early Discharge (Postoperative Day < 2)

| **Parameter** | **Procedure** | **SSN Pathway (No Penalty)** | **ERAS Pathway (Before Penalty)** | **ERAS Pathway (With 30% DRG Penalty)** | **Δ ERAS vs SSN (With Penalty)** | **p-value** |
| --- | --- | --- | --- | --- | --- | --- |
| **Cases affected by penalty (%)** | Hemithyroidectomy | 0% | 100% | 100% | All ERAS penalized, SSN unaffected | — |
|  | Total thyroidectomy | 0% | 92% | 92% | Vast majority of ERAS penalized | — |
| **DRG reimbursement (€)** | Hemithyroidectomy | 3,420 ± 210 | 3,420 ± 210 | 2,394 ± 185 (−30.0%) | −1,026 ± 60 vs SSN | < 0.001 |
|  | Total thyroidectomy | 4,780 ± 250 | 4,780 ± 250 | 3,346 ± 230 (−30.0%) | −1,434 ± 75 vs SSN | < 0.001 |
| **Variable cost (€)** | Hemithyroidectomy | 2,220 ± 150 | 1,860 ± 130 | 1,860 ± 130 | −360 ± 80 vs SSN | 0.001 |
|  | Total thyroidectomy | 3,000 ± 200 | 2,520 ± 190 | 2,520 ± 190 | −480 ± 95 vs SSN | 0.002 |
| **Length of stay (days)** | Hemithyroidectomy | 2.3 ± 0.6 | 1.1 ± 0.3 | 1.1 ± 0.3 | −1.2 ± 0.4 vs SSN | < 0.001 |
|  | Total thyroidectomy | 3.4 ± 0.9 | 1.6 ± 0.5 | 1.6 ± 0.5 | −1.8 ± 0.6 vs SSN | < 0.001 |
| **Contribution margin (€)** | Hemithyroidectomy | 1,200 ± 180 | 1,560 ± 175 | 534 ± 165 | +55 ± 75 vs SSN | < 0.001 |
|  | Total thyroidectomy | 1,780 ± 210 | 1,780 ± 205 | −(approx.) | −610 ± 110 vs SSN | < 0.001 |
| **Incremental ERAS margin vs SSN (€)** | Hemithyroidectomy | +260 ± 85 (base case) | — | +55 ± 75 (with penalty) | Strongly attenuated | < 0.001 |
|  | Total thyroidectomy | −190 ± 95 (base case) | — | −610 ± 110 (with penalty) | Gap widened vs SSN | < 0.001 |
| **Aggregate effect (hospital level)** | Both procedures | Reference (indexed base case) | — | 14.8% reduction in total contribution margin | Net loss despite lower resource use | 0.002 |

## Supplementary Table. 12 Item‑level unit costs by perioperative dominion (2025 euros)

| **Dominion** | **Item / resource** | **Unit (per)** | **Unit cost (€)** | **Data source** | **Used in model component** |
| --- | --- | --- | --- | --- | --- |
| **Preoperative dominion** | Preoperative endocrinology / surgical outpatient visit | Visit | 85.00 | Outpatient tariff schedule (OUT‑001) | Baseline preoperative assessment |
|  | Preoperative thyroid function tests (TSH, FT4, FT3) | Panel | 16.00 | Laboratory tariff schedule (LAB‑010) | Routine preoperative blood tests |
|  | Preoperative neck ultrasound | Exam | 70.00 | Radiology tariff (RAD‑010) | Standard staging / planning |
|  | Anaesthesiology pre‑operative assessment | Visit | 60.00 | Internal cost‑accounting (ANA‑001) | Pre‑operative risk evaluation |
| **Intraoperative dominion** | Operating room time (staff + overhead) | 60 minutes | 520.00 | Theatre cost‑accounting (OR‑001) | Base operative cost per hour |
|  | Intraoperative neuromonitoring (IONM) system use | Case | 110.00 | Amortized capital + disposables (OR‑IONM‑001) | Nerve monitoring during thyroidectomy |
|  | IONM disposable tube/electrodes | Set | 65.00 | Procurement registry (MED‑IONM‑SET) | Single‑use consumables for IONM |
|  | Energy‑based device (EBD) use | Case | 160.00 | Device contract / procurement (OR‑EBD‑001) | Vessel sealing and hemostasis |
|  | EBD disposable handpiece | Piece | 140.00 | Procurement registry (MED‑EBD‑HP) | Single‑use handpiece |
|  | Intraoperative PTH (ioPTH) assay | Test | 28.00 | Laboratory tariff (LAB‑020) | Rapid PTH measurement in selected cases |
|  | Autofluorescence device for parathyroid detection | Case | 95.00 | Amortized capital + single‑use components (OR‑AF‑001) | Intraoperative parathyroid identification |
|  | Autotransplantation set / sutures for parathyroid | Case | 18.00 | Procurement registry (SURG‑SET‑001) | Parathyroid autotransplantation when required |
| **Discharge dominion** | Discharge planning / education session | Session | 25.00 | Nursing time + materials (WARD‑EDU‑001) | ERAS discharge counseling and instructions |
|  | Preparation of discharge documents and prescriptions | Case | 10.00 | Administrative cost estimate (ADMIN‑001) | Final discharge processing |
|  | Post‑discharge telephone follow‑up call | Call | 8.00 | Nurse/physician time (TELE‑001) | Structured ERAS follow‑up within 48–72 hours |
| **Postoperative dominion** | Nursing care and ward overhead | Bed‑day | 190.00 | Internal cost‑accounting, variable component (WARD‑001) | Variable portion of bed‑day cost |
|  | Ward consumables (dressings, sets, syringes) | Bed‑day | 22.00 | Aggregated procurement registry (WARD‑002) | Routine consumables per inpatient day |
|  | Analgesics (paracetamol IV/PO) | Dose | 1.20 | Pharmacy registry (MED‑001) | Post‑operative analgesia per inpatient day |
|  | Proton‑pump inhibitor | Dose | 0.90 | Pharmacy registry (MED‑002) | Gastric prophylaxis per inpatient day |
|  | Antiemetics | Dose | 1.50 | Pharmacy registry (MED‑003) | As‑needed antiemetics, averaged per day |
|  | LMWH prophylaxis | Dose | 3.80 | Pharmacy registry (MED‑004) | Thromboprophylaxis per inpatient day |
|  | Basic metabolic panel | Panel | 9.50 | Laboratory tariff (LAB‑001) | Routine daily labs during inpatient stay |
|  | Serum calcium | Test | 4.20 | Laboratory tariff (LAB‑002) | Early post‑operative calcium monitoring |
|  | PTH assay | Test | 18.00 | Laboratory tariff (LAB‑003) | Early post‑operative PTH testing |
|  | Emergency department visit | Visit | 120.00 | Hospital accounting / regional tariff (ED‑001) | Unplanned post‑discharge ED contacts |
|  | Neck ultrasound (post‑operative, if needed) | Exam | 70.00 | Radiology tariff (RAD‑001) | Post‑operative imaging in selected cases |
|  | Oral calcium supplement | Treatment course | 12.00 | Pharmacy registry (MED‑005) | Management of post‑operative hypocalcemia |
|  | Vitamin D supplement | Treatment course | 8.00 | Pharmacy registry (MED‑006) | Management of post‑operative hypocalcemia |
